# Evaluation of Microleakage and Marginal Ridge Fracture Resistance of Primary Molars Restored with Three Restorative Materials: A Comparative *in vitro* Study

**DOI:** 10.5005/jp-journals-10005-1294

**Published:** 2015-08-11

**Authors:** Tapan Satish Yeolekar, Nagalakshmi Ramesh Chowdhary, KS Mukunda, NK Kiran

**Affiliations:** Postgraduate Student, Department of Pedodontics, Sri Siddhartha Dental College and Hospital, Tumkur, Karnataka, India; Professor and Head, Department of Pedodontics, Sri Siddhartha Dental College and Hospital, Tumkur, Karnataka, India; Professor, Department of Pedodontics, Sri Siddhartha Dental College and Hospital, Tumkur, Karnataka, India; Reader, Department of Pedodontics, Sri Siddhartha Dental College and Hospital, Tumkur, Karnataka, India

**Keywords:** Compomer, Dye penetration, Fracture resistance, Microleakage, Packable composite resin, Silorane composite resin.

## Abstract

Composite restorations are popular because of their superior esthetics and acceptable clinical performance. But shrinkage is still a drawback. Polymerization shrinkage results in volumetric contraction, leading to deformation of the cusps, microleakage, decrease of marginal adaptation, enamel micro-cracks and postoperative sensitivity.

A new class of ring opening resin composite based on silorane chemistry has been introduced with claims of less than 1% shrinkage during polymerization. The present study was conducted to evaluate and compare the ability of low shrink silorane based material, a packable composite and a compomer to resist microleakage in class II restorations on primary molars and evaluate marginal ridge fracture resistance of these materials.

Sixty human primary molars were selected. Class II cavities were prepared and the teeth were divided into three groups of twenty each. Groups were as follows group I: low shrink composite resin (Filtek P90). Group II: packable composite (Filtek P60) and Group III: compomer (Compoglass F). Half of the teeth were used for microleakage and the rest for marginal ridge fracture resistance. For microleakage testing, dye penetration method was used with 1% methylene blue dye. Followed by evaluation and grading under stereomicroscope at 10* magnification. Fracture resistance was tested with universal testing machine.

It was concluded that low shrink silorane based composite resin showed the least amount of microleakage, whereas compomer showed the highest microleakage. Packable composite resisted fracture of marginal ridge better than other composite resins. Marginal ridge fracture resistance of packable composite was comparable to the intact side.

**How to cite this article:** Yeolekar TS, Chowdhary NR, Mukunda KS, Kiran NK. Evaluation of Microleakage and Marginal Ridge Fracture Resistance of Primary Molars Restored with Three Restorative Materials: A Comparative *in vitro* Study. Int J Clin Pediatr Dent 2015;8(2):108-113.

## INTRODUCTION

In recent years, resin-based composite materials have been widely used in restorative dentistry. The popularity of these restorations has increased because of a demand for cosmetic, tooth-colored restorations and a decreased acceptance of traditional amalgam by the patients. Resin composites have improved greatly since their introduction and are now the materials of choice for most of the restorations. Despite recent dramatic improvements in the technology of composite resins and their adhesive systems, polymerization shrinkage, which occurs as the material cures, remains a major problem. This shrinkage pulls the restorative material away from the cavity walls, resulting in rupture of the adhesion and the formation of marginal gaps. These gaps cause postoperative sensitivity, discoloration and secondary caries at the restoration interface, and pulpal pathology, eventually leading to failure of the restorations.^1^

Compomers have shown better physical properties to those of light hardened glass ionomer cements such as adhesion to tooth substance, fluoride release and biocompatibility. ^2^

The packable composites are indicated for stress bearing posterior restorations with improved handling characteristics and with an application technique similar to amalgam.^3^

Recently, a new composite resin Filtek P90 has been developed. It uses blocks of siloxanes and oxiranes to provide a biocompatible, hydrophobic, low-shrinking silorane as base. In these resins, polymerization takes place by cationic ‘ring-opening’ mechanism resulting in minimal polymerization shrinkage of less than 1%.^4^ It reduces the disadvantages faced during use of meth-acrylate based material.

Hence, the aim of present study was to evaluate and compare the ability of low shrink silorane based material, a packable composite and a compomer to resist microleakage in class II restorations on primary molars and to evaluate marginal ridge fracture resistance of these materials. The research hypothesis was that no difference in microleakage and marginal ridge fracture resistance of primary molars restored would be observed with different resin systems.

## MATERIALS AND METHODS

### Collection of Sample

Sixty human primary molars were randomly selected for the study. Teeth without any visible structural defects or previous restorations were selected.

### Cavity Preparation

**Fig. 1:**
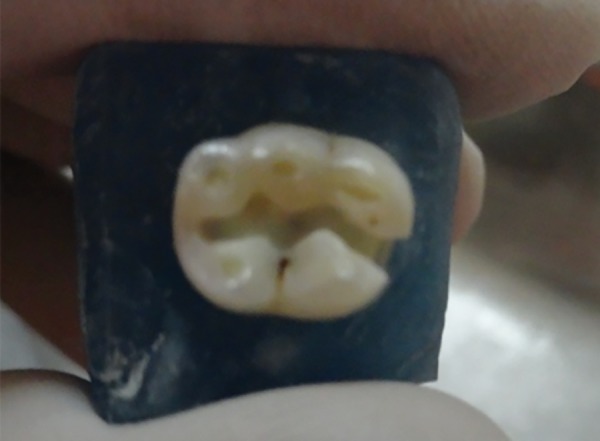
Cavity preparation

Class II cavities were prepared and teeth were divided into three groups of twenty each (Fig. 1). Class II cavity was prepared by removing all carious tooth structure and undermined enamel using diamond bur (Mani DIA-BUR ex-41) in contra angled airotar handpiece (NSK, Japan) with water coolant. Medium sized cavites of approximately 4 mm mesiodistally from the proximal surface of primary molars were made. The isthmus of cavity was approximately 2/3rd of buccolingual width, using inter-cuspal distance as reference. The bur was replaced after every five preparations. Teeth were divided into three groups, group I consisted of teeth restored with low shrink composite resin (Filtek P90) (3M ESPE,MN, USA). Group II consisted of teeth restored with packable composite (Filtek P60) (3M ESPE,MN, USA) and group III consisted of teeth restored with compomer Compoglass F (Ivoclar Vivadent).

### Restorative Procedure

**Fig. 2:**
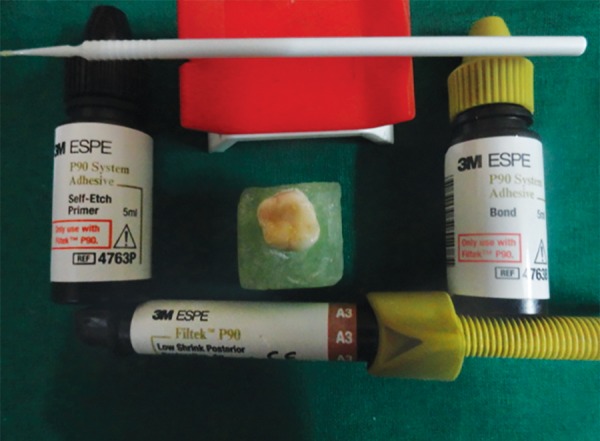
Filtek P90 and bonding system used

*For group I:* Filtek P90 self-etch primer (3M ESPE,MN, USA) (Fig. 2) was applied to the prepared cavity for 15 seconds and was light cured for 10 seconds. Then the P90 bond was applied, air dried to a homogenous film and was light cured for 10 seconds with LED light curing unit. The silorane composite was then placed in the cavity using oblique incremental technique and each increment was cured for 40 seconds.

**Fig. 3:**
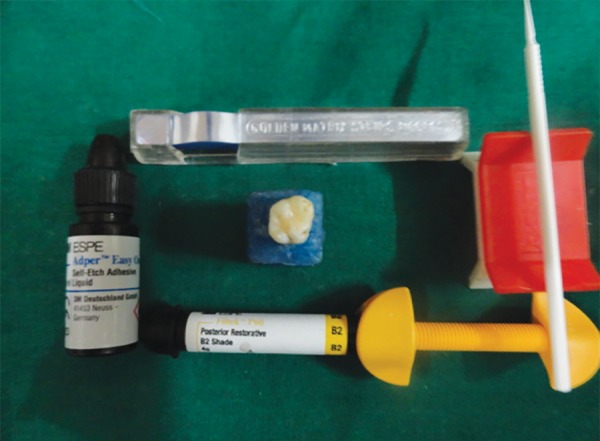
Filtek P60 and bonding system

*For group II:* Self-etch adhesive Adper Easy One (3M ESPE, Germany) (Fig. 3) was applied to the prepared cavity air dried for 5 seconds and light cured for 10 seconds. Resin composite Filtek P60 was placed in the cavity using oblique incremental technique and each increment was cured with LED light curing unit for 40 seconds.

**Fig. 4:**
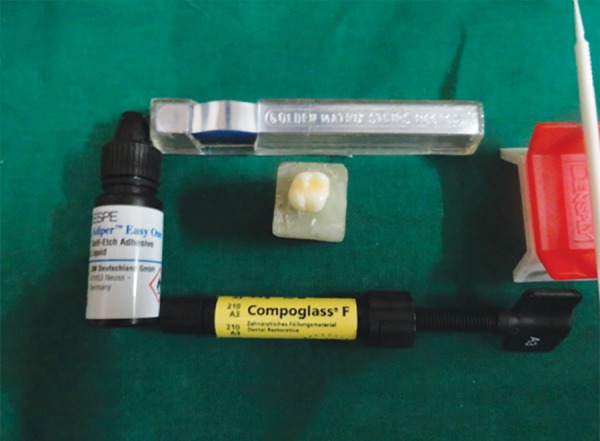
Compoglass F and bonding system

*For group III:* Self-etch adhesive Adper Easy One (3M ESPE, Germany) (Fig. 4) was applied to the prepared cavity air dried for 5 seconds and light cured for 10 seconds. Resin composite Compoglass F was placed in the cavity using oblique incremental technique and each increment was cured with LED light curing unit for 40 seconds.

### Thermocycling

Following restorations of the prepared cavities the excess composite was removed and finishing and polishing was done using composite finishing kit OptraPol (Ivoclar Vivadent Germany). Teeth were thermocyled 5° to 55°C (± 2°C) for 200 cycles with a dwell time of 15 seconds and a transfer time of 1 minute. Half of the teeth were used for microleakage and rest for marginal ridge fracture resistance.

### Microleakage Testing

**Fig. 5:**
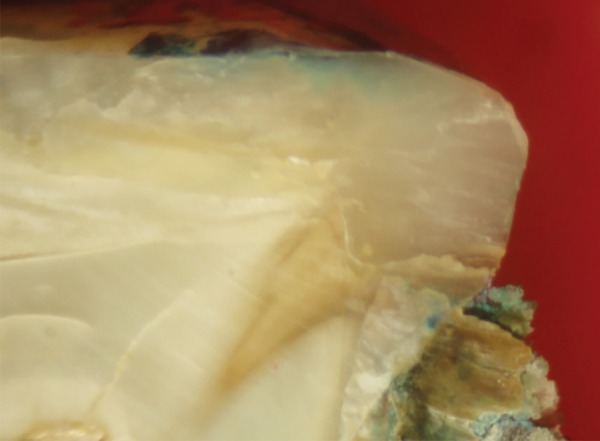
Score 0

**Fig. 6:**
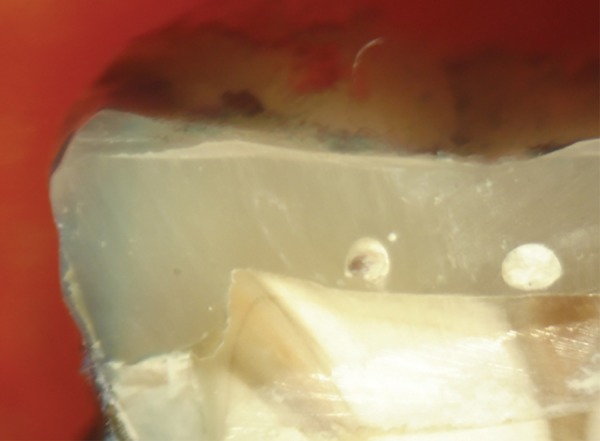
Score 1

**Fig. 7:**
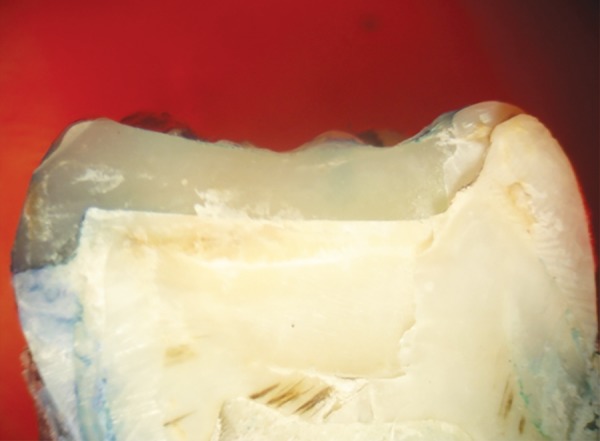
Score 2

**Fig. 8:**
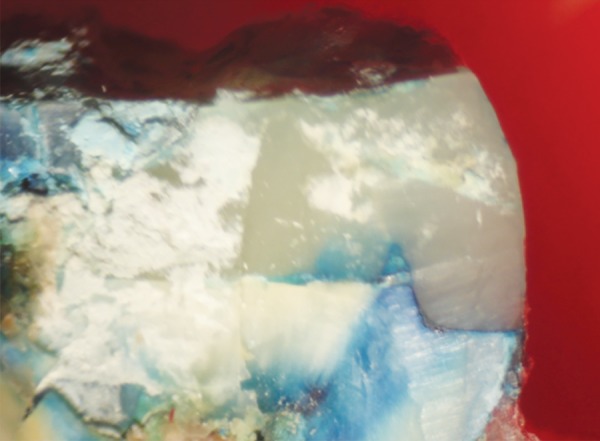
Score 3

Ten teeth from each experimental groups I, II and III were randomly selected for microleakage test. All teeth received two coats of nail polish on the entire tooth surface except for the restoration and a 2 mm rim of tooth structure around the restoration and allowed to air dry. All the teeth were immersed in 1% methylene blue dye for 24 hours. After 24 hours, teeth were washed in tap water. This was followed by mesiodistal sectioning of teeth in two sections using diamond disk. Stereomicroscope (Olympus) magnatus at 10× magnification was used to evaluate the amount of microleakage. Scores from 0 to 3 (Figs 5 to 8) were assigned depending upon the amount of dye penetration (Table 1).

**Table 1:** **Table 1:** Scoring criteria used^14^

*Score*		*Criteria*	
0		No dye penetration	
1		Dye penetration into half extension of the cervical wall	
2		Dye penetration into complete extension of the cervical wall	
3		Dye penetration into the cervical and axial walls toward the pulp	

### Evaluation of Fracture Resistance

Half of the teeth from each group were evaluated for marginal ridge fracture resistance testing using universal testing machine (Llyod LR- 50 K, USA). The intact opposite side marginal ridges were also subjected to fracture resistance testing. The load at which marginal ridges fractured indicated fracture resistance in Newtons (N).

## RESULTS

Data were analyzed using SPSS 17.0 software.

Microleakage

**Graph 1:**
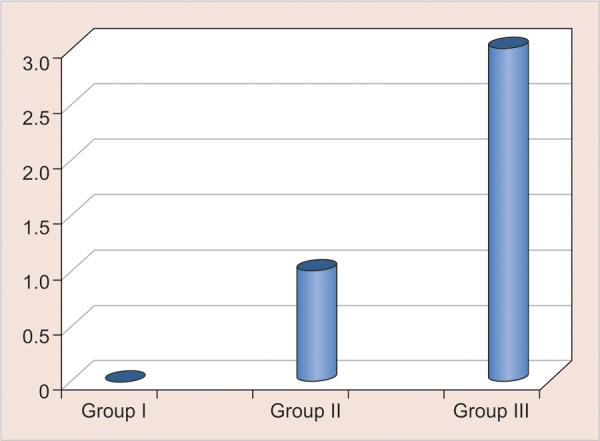
Median microleakage scores obtained: group I Filtek P90; group II Filtek P60; group III Compoglass F

Median scores at 50th and 75th percentile (Table 2 Graph 1) were calculated using descriptive statistics and the groups were compared using Kruskal-Wallis H test (Table 3).

The p-value was taken significant when less than 0.05. Kruskal-Wallis H test indicated significant difference in the microleakage scores among the various materials studied. Kruskal-Wallis test was followed by Mann-Whitney U test for intergroup comparison was carried out. Group I Filtek P90 with median score 0 (0―0.75) was found to be highly significant among all groups.

Marginal ridge fracture resistance

For marginal ridge fracture resistance post hoc Tukey test was used, it was found that there is significant difference between the two materials: group I Filtek P90 and group II Filtek P60.

**Graph 2:**
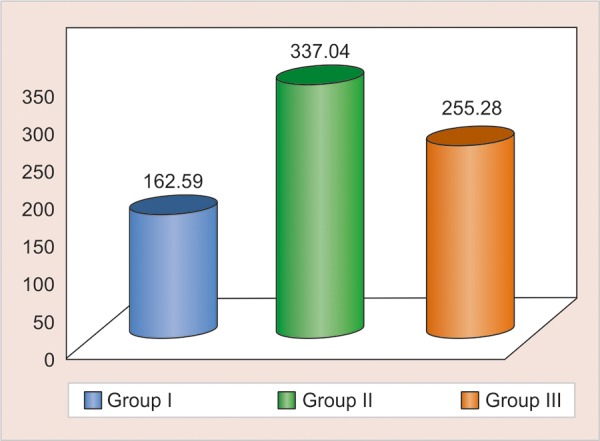
Comparative mean for all groups for fracture resistance (Restored side): group I Filtek P90; group II Filtek P60; group III Compoglass F

**Table 2:** **Table 2:** Median microleakage scores at 50th and 75th percentile

*Sl. no.*		*Groups*		*Number (n)*		*Median*		*25th percentile*		*75th percentile*	
1		1 Filtek P90		10		0		0		0.75	
2		II Filtek P60		10		1		1		1.75	
3		III Compoglass F		10		3		2		3	

**Table 3:** **Table 3:** Comparison among the three groups for microleakage using Kruskal-Wallis test

*Sl. no.*		*Material*		*Number (n)*		*Median*		*Mean ranks*		*H-value*		*p-value*	
1		Group I Filtek P90		10		0		7.9		15.3		0.0005, highly	
2		Group II Filtek P60		10		1		15.4		―		significant	
3		Group III Compoglass F		10		3		23.3		―		―	

**Table 4:** **Table 4:** Comparison among three groups for fracture resistance (intact side) using one-way ANOVA

*Sl. no.*		*Material*		*Number (n)*		*Mean*		*Standard deviation*		*Standard error*		*One-way ANOVA*	
1		Filtek P90		10		332.22		104.97		33.19		F -3.06,	
2		Filtek P60		10		403.75		121.09		38.29		p-value -0.0634, not	
3		Compoglass F		10		479.93		166.80		52.74		significant	

One-way ANOVA (Table 4) for intact sides of all the three groups showed no significant difference in fracture resistance thus all sides had more or less same strength, with F -3.06, p value -0.0634.

Mann-Whitney U test was carried out for inter group comparison between restored marginal ridges of all the three groups. Graph 2 shows comparative mean of all groups (restored side) for fracture resistance. Z-value and p-value obtained by Mann-Whitney U test is tabulated (Table 5). Filtek P60 mean scores of 337.04 (SD - 121.3) was found to be having better resistance to fracture.

Mann-Whitney U test was used for intra group comparison between the restored marginal ridges and the intact sides within a same group. Z-value and p-value obtained for all three groups is tabulated. It was found that fracture resistance of group II was comparable with intact side marginal ridge (Table 6).

## DISCUSSION

Dental caries has long been recognized as an infectious disease requiring a susceptible host, a cariogenic microbial flora, and a diet high in refined carbohydrate to sustain that flora. Practically, there is no geographic area in the world whose inhabitants do not exhibit any evidence of dental caries.^5^

Composite resins have been successfully used for dental restoration for over 50 years but polymerization shrinkage is still the major drawback. Polymerization shrinkage results in volumetric contraction, causing stresses in bonded restorations that can lead to deformation of the cusps, microleakage, decrease of marginal adaptation, enamel microcracks and postoperative sen-sitivity.^6-8^

Microleakage at the restoration tooth interface has been identified as a cause of secondary caries and postoperative sensitivity. It is generally agreed that microleakge is common to nearly all restorative materials and techniques.^9^

**Table 5:** **Table 5:** Intergroup comparison for fracture resistance on restored side using Mann-Whitney U test

*Pairwise comparison*		*Mean ranks*		Z-value		p-value		Significance	
Group I		6		3.36		0.0004		HS	
Group II		15							
Group I		6.4		3.06		0.0011		HS	
Group III		14.6							
Group III		12.6		1.55		0.1211		NS	
Group II		8.4							

HS: Highly significant; NS: Not significant

**Table 6:** **Table 6:** Intragroup comparison for fracture resistance using Mann-Whitney U test

*Pairwise comparison*		*Mean ranks*		Z value		p value		Significance	
Group I				3.36		0.0004		HS	
Restored		6							
Intact		15							
Group II				1.13		0.1292		NS	
Restored		9							
Intact		12.1							
Group III				3.36		0.0004		HS	
Restored		6							
Intact		15							

HS: Highly significant; NS: Not significant

Microleakage and marginal ridge fracture resistance test have been mainly carried out on permanent teeth hence in present study 60 primary molars were chosen to check for any variability in these teeth.

As self-etch adhesive is provided with silorane based resin composites by the manufacturer. So in order to keep the bonding system constant, Adper Easy One which is a self-etch adhesive was used for methacrylate resin composite as well as compomer in the present study.

Many authors have preferred metal matrix band for the restoration of class II composite because they can be better contoured than a clear polyester matrix. In the present study mylar strip matrix band was used to restore proximal box of class II cavities to assess whether any variation exists in microleakage and marginal ridge fracture resistance of primary molars.

One of method to minimize polymerization stress is by altering the C-factor, which also depends on placement technique. Small increments with greater free surfaces in lieu of bonded ones would compensate for polymerization stress rendering a better integration between the composite and tooth structure, thus resulting in a better sealed restoration and limits the development of contraction forces between opposing walls, reducing stress build up and gap formation.^10^ Thus in the present study, oblique incremental technique was used.

The results of *in vitro* microleakage studies are close to clinical reality, because human teeth and clinical protocols are used. Results of the present study were in agreement with many previous studies^11-17^ which showed that microleakage of low shrink silorane based resin had lesser polymerization shrinkage. The probable reason for less polymerization shrinkage and therefore lesser microleakage can be attributed to silorane system which uses ‘ring opening polymerization’ instead of free radical polymerization of dimethacrylate monomers used in groups II and III.

It was found that there was statistical significant difference among the three groups in marginal ridge fracture resistance of restored side, whereas there was no statistical significant difference among the three groups in fracture resistance of intact side. Thus, intact side marginal ridge for all three groups had similar strength. This was in accordance with previous study done by Prabhu et al.^18^

When group I was compared with group II, it was found that group II had higher fracture resistance. This was in accordance to previous studies.^19,20^

In a comparison between groups I and III, III found to be better. This was in contradiction to previous study which showed that compomers have lowest fracture resistance attributing to its lowest percentage of fillers by volume. Also, presence of ion-leachable glass powder may disharmonize the critical filler content.^21^

The probable reason for lesser fracture resistance of group I in present study could be attributed to lesser degree of subsurface polymerization of silorane composites as compared to compomers which undergo free radical type of polymerization reaction.

In the inter group comparison between group II Filtek P60 with group III Compoglass F, No material was found to be statistically significant, thus both the groups have similar fracture resistance. This was in contradiction to previous study done by Yap et al who found that there is significant difference in the two materials.^20^

Thus, it was proved in this study that low shrink silorane based resins are best in terms of microleakage as compared to currently used materials in restoration of class II cavities of primary teeth. Packable composites were superior to other types of composites with respect to marginal ridge fracture resistance. Additional *in vivo* studies with larger sample size, should be done for evaluating the long term clinical performance, and to further insight into the efficiency of the restorative materials in class II cavity preparations of primary molars.

## CONCLUSION

- Microleakage is inevitable irrespective of type of material being used.
- Low shrink silorane based composite resin showed least microleakage, followed by packable composite, whereas compomer showed highest microleakage among the three groups.
- Packable composite resisted fracture of marginal ridge better than low shrink silorane based composite resin. Fracture resistance of packable composite was comparable to compomer. Marginal ridge fracture resistance of materials was in order as follows group II = group III > group I.
- Marginal ridge fracture resistance of packable composite (Group II Filtek P60) was comparable to that of the intact side marginal ridge.
